# Electrostatic Control of Electronic Structure in Modular Inorganic Crystals

**DOI:** 10.1021/jacs.4c13637

**Published:** 2024-12-19

**Authors:** Kanta Ogawa, Aron Walsh

**Affiliations:** Department of Materials, Imperial College London, London SW7 2AZ, U.K.

## Abstract

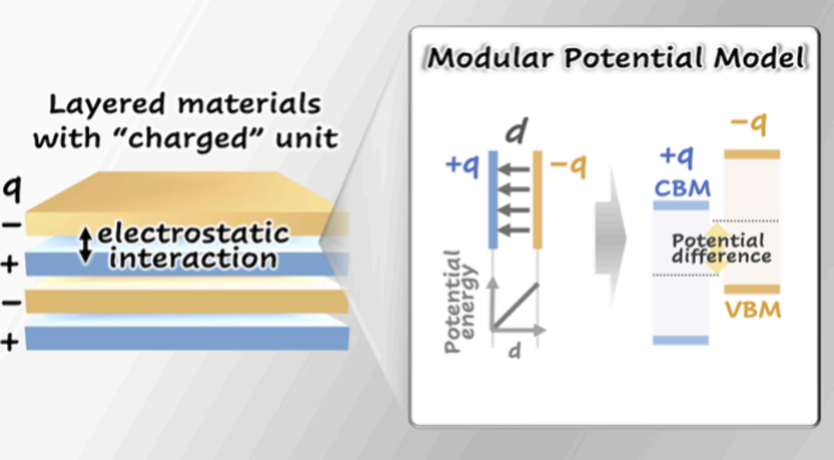

The rules that govern structure and bonding, established for elemental solids and simple compounds, are challenging to apply to more complex crystals formed of polyatomic building blocks, such as layered or framework materials. Whether these modular building blocks are electrically neutral or charged influences the physical properties of the resulting crystal. Despite the prevalence of alternating charged units, their effects on the electronic structure remain unclear. We demonstrate how the distribution of charged building blocks, driven by differences in the electrostatic potential, governs the electronic band energies formed in layered crystals. This coarse-grained model predicts the spatially separated valence and conduction band edges observed in the metal-oxyhalide Ba_2_Bi_3_Nb_2_O_11_Cl and explains observed property trends in the Sillén–Aurivillius crystal system. Moreover, the general nature of the model allows for extension to other modular structure types, illustrated for Sillén and Ruddlesden–Popper layered compounds, and can support the rational design of electronic properties in diverse materials.

## Introduction

The structures and properties of many types of compounds can be readily described in terms of interatomic interactions, from the geometric considerations that underpin the radius ratio rules^1,2^ to the electronegativity scales that inform the degree of ionicity of chemical bonds.^3^ However, there are also many classes of materials that may be better represented as architectures of coarse-grained polyatomic modules with various scales from 2D planes to 1D channels and 0D clusters. Not only the orbital interactions within each module but also the interaction between modules determines the resulting electronic structure and physical properties. Such a modular perspective helps us design crystal structures in a chemically intuitive way,^4,5^ with significant recent progress in the exploration of novel complex structure types^6,7^ and superatomic solids.^8,9^

**Figure 1:**
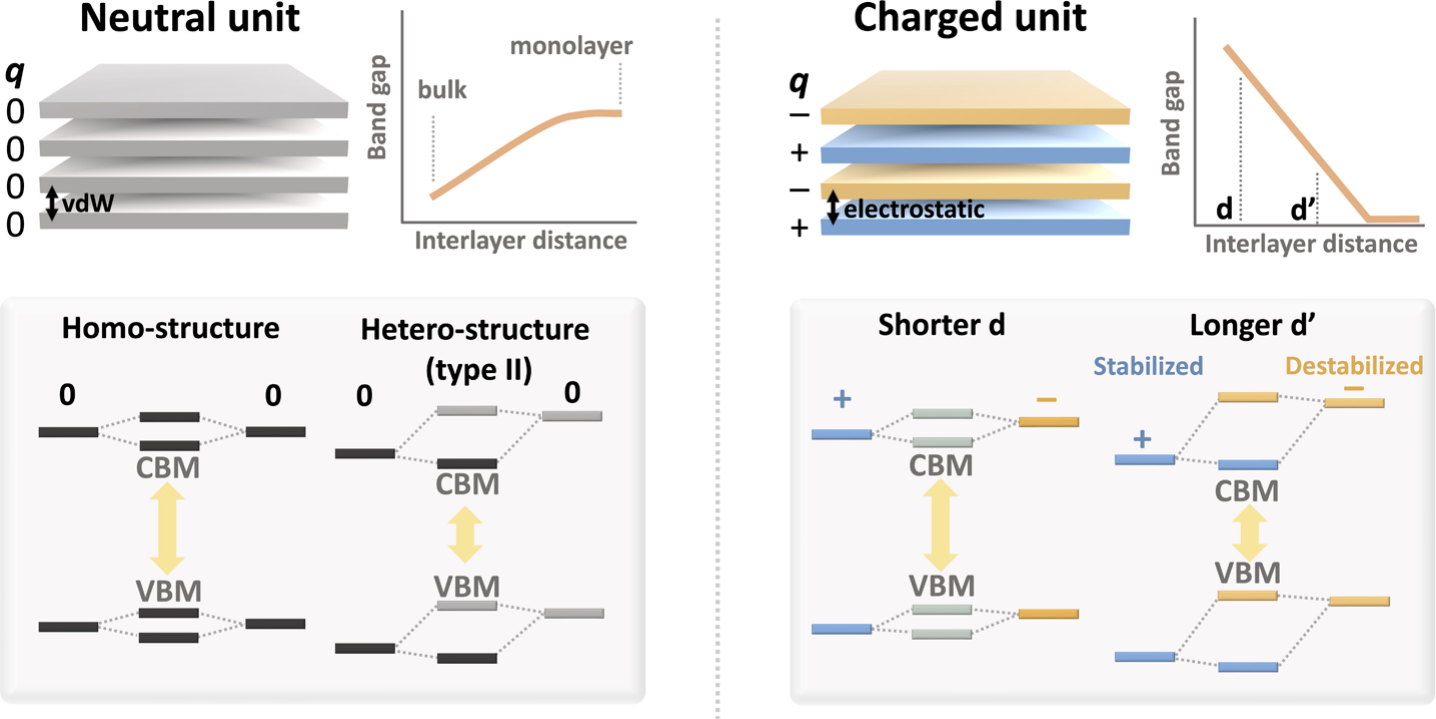
Illustration of the stacking sequence of neutral and charged units in layered modular crystals. The relationship between the band gap and interlayer distance is based on the consideration of the underlying orbital interactions in the neutral and charged modular materials. The type II band offset refers to the spatial separation of the valence band maximum (VBM) and conduction band minimum (CBM) wave functions between two building blocks.

Modular building units of crystals can be categorized as electrically neutral or charged.^10^ “Neutral” assemblies are connected by van der Waals (vdW) interactions between building blocks as found in materials such as graphene, phosphorus, and transition metal dichalcogenides (TMDs). Here the integrated sum of the nuclear charge (Z) and electron density (ρ) of each building block is 0, e.g., the repeating layers in MoS_2_. The electronic structure of their stacking compounds can usually be approximated from the band edge positions of the isolated building block. In a homostructure, layers at the same electrochemical potential interact, where increasing interlayer distance or decreasing layer number enlarges the band gap toward that of the monolayer (Figure 1).^11,12^ In a heterostructure, the band gaps are depicted as a superposition of the original monolayers, where the highest valence and lowest conduction band edge among the original units determines the band edge positions.^13,14^ This can give rise to straddling (type I), staggered (type II), and broken band gap (type III) behavior.^15^ Weak interlayer interactions provide a fascinating playground for novel physics,^13^ including magic-angle bilayers of graphene^16,17^ and Moiré excitons in TMDs.^18−20^

Assemblies carrying a net charge result in an electrostatic potential difference between building blocks, providing another controlling parameter for their electronic structures.^21^ Here, the total charge of the module is obtained by integrating the charge within the building block such as the [LiAl_2_(OH)_6_]^+^ layers in cookeite silicate minerals.^22^ For ionic solids, the electrostatic potentials of each atomic site are often described with the Madelung model by regarding the constituent cations/anions as fixed point charges with long-range interactions accounting for using an appropriate summation method over periodic boundary conditions.^23^ Another common assembly is a 2D plane.^10,24^ The interlayer electric field between oppositely charged planes explains the polar discontinuity and the quasi-2D electron gas at the LaAlO_3_/SrTiO_3_ interface, where they are formally divided to planar [AlO_2_]^−^, [LaO]^+^, [TiO_2_]^0^, [SrO]^0^ building blocks, again within an ionic approximation but without considering the detailed nature of the bonding.^25,26^

There is a large family of layered modular materials with thicker units, where electrostatic factors are less explored. One case is the Sillén–Aurivillius layered oxyhalides, which have emerged as ferroelectric materials,^27^ oxide-ion conductors,^28^ and visible-light photocatalysts.^29−31^ Layered stacking sequence of Bi/Pb oxide-based fluorite-like [A_2_O_2_], halide [X], and perovskite [A′_*n*–1_B_*n*_O_3*n*+1_] composes the general formula [A_2_O_2_][X][A_2_O_2_][A′_*n*–1_B_*n*_O_3*n*+1_]. Their electronic structure features a valence band maximum (VBM) and conduction band minimum (CBM) on different layers,^30,32^ providing a spatial separation of photoexcited charge carriers.^32−34^ Such a spatially indirect band offset is desirable for solar-to-energy conversion systems requiring long-lived charge carriers.^35,36^ Revealing the origin of these unique characteristics will enable better control of the band gaps and charge separation properties.

In this study, we demonstrate that internal electric fields between charged blocks can be used to describe the electronic structures of chemically complex modular materials, such as layered metal oxyhalides. We introduce a coarse-grained electrostatic model that successfully reproduces trends in varying the layer charge and spatial separation. The description of charged building blocks is independent of the polarity of the underlying local interatomic interactions. The modular potential model is demonstrated to describe the electronic structures and chemical trends in other layered materials.

## Results and Discussion

### Spatially Separated Band Edges in Oxyhalides

**Figure 2:**
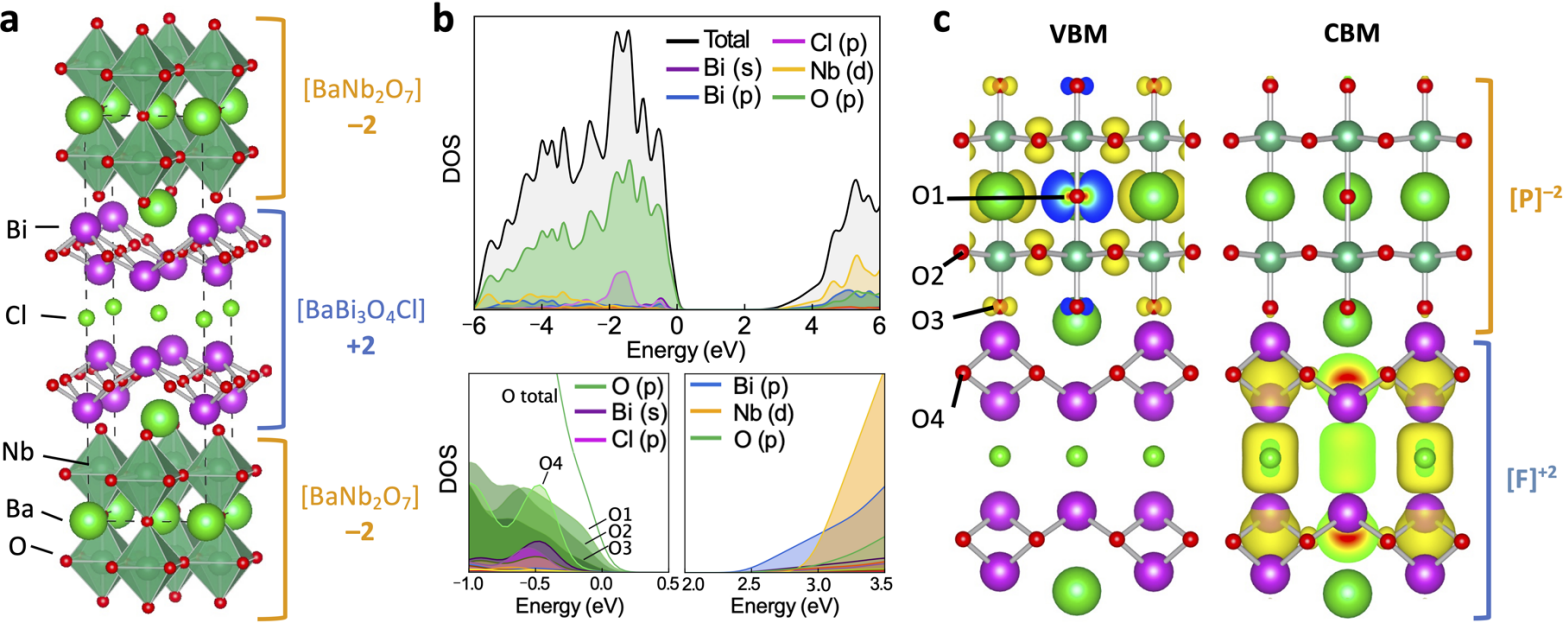
(a) Crystal structure of Ba_2_Bi_3_Nb_2_O_11_Cl described as the stacking of [Ba_1_Bi_3_O_4_Cl]^+2^, [BaNb_2_O_7_]^−2^ blocks. (b) Electronic density of states (HSE06/DFT), with valence band maximum (VBM) from the perovskite block and the conduction band minimum (CBM) from the fluorite block. (c) Electron density map of the valence band maximum and conduction band minimum at an isosurface value of 1 × 10^–4^ and 2 × 10^–5^ e/Å^3^, respectively.

Among Sillén–Aurivillius oxyhalides, Ba_2_Bi_3_Nb_2_O_11_Cl (*P*4/*mmm*) with double perovskite layer (*n* = 2) was chosen as a model case owing to its relative simplicity and wide application range.^37,38^ This material consists of a stacking sequence of the positive [fluorite + halide] block [BaBi_3_O_4_Cl]^+2^ and the negative perovskite block [BaNb_2_O_7_]^−2^ (Figure 2a). Each block is a common module in other layered materials,^39,40^ and the Coulomb interaction drives the alternative stacking with opposite charges and close subcell parameters.^41^ The fluorite and perovskite blocks are described as [F]^+2^, and [P]^−2^, respectively.

The electronic structure was obtained using density functional theory (DFT) with the Heyd–Scuseria–Ernzerhof (HSE06) hybrid exchange-correlation functional,^42^ as implemented in the Vienna ab initio simulation package (VASP).^43^ The calculated lattice parameters (*a* = 5.61, *c* = 18.93 Å) and band gap (2.45 eV) are in good agreement with previous experimental values (*a* = 5.63, *c* = 18.82 Å, *E*_g_ = 2.63 eV).^38,40^Figure 2b shows the calculated electronic density of states (DOS) of Ba_2_Bi_3_Nb_2_O_11_Cl. The upper part of the valence band is composed of O 2p orbitals in the [P]^−2^ block with the contribution order of O1 > O2 > O3 from the middle of the block to the interface with [F]^+2^ (Figure 2b,c). The lower part of the conduction band is from the Bi 6p orbitals in the [F]^+2^ block. The interlayer Bi interaction within [F]^+2^ block across the halide layer contributes to the highly dispersed nature of the conduction band.^38^ The spatially separated upper valence band on a perovskite block and lower conduction band on a Bi-based fluorite block is common in other Sillén–Aurivillius compounds, and is considered to be a key for efficient charge separation and high photocatalytic activity.^32,44^

### Interlayer Distance Effect

Spatially separated band edges are attractive for both solar-to-energy conversion and exciton physics, as extensively studied in type-II heterostructures of TMDs.^14^ In such cases, one can directly estimate the band energies from the superpositions of the original TMD band edges (Figure 1). However, this cannot be applied to the present case. The valence band energy (ionization potential) of Bi-based materials is usually higher than conventional oxides (e.g., Nb-based) due to filled Bi 6s–O 2p antibonding states.^45,46^ A simple superposition of the component orbitals cannot reproduce the observed order in Figure 2b.

**Figure 3:**
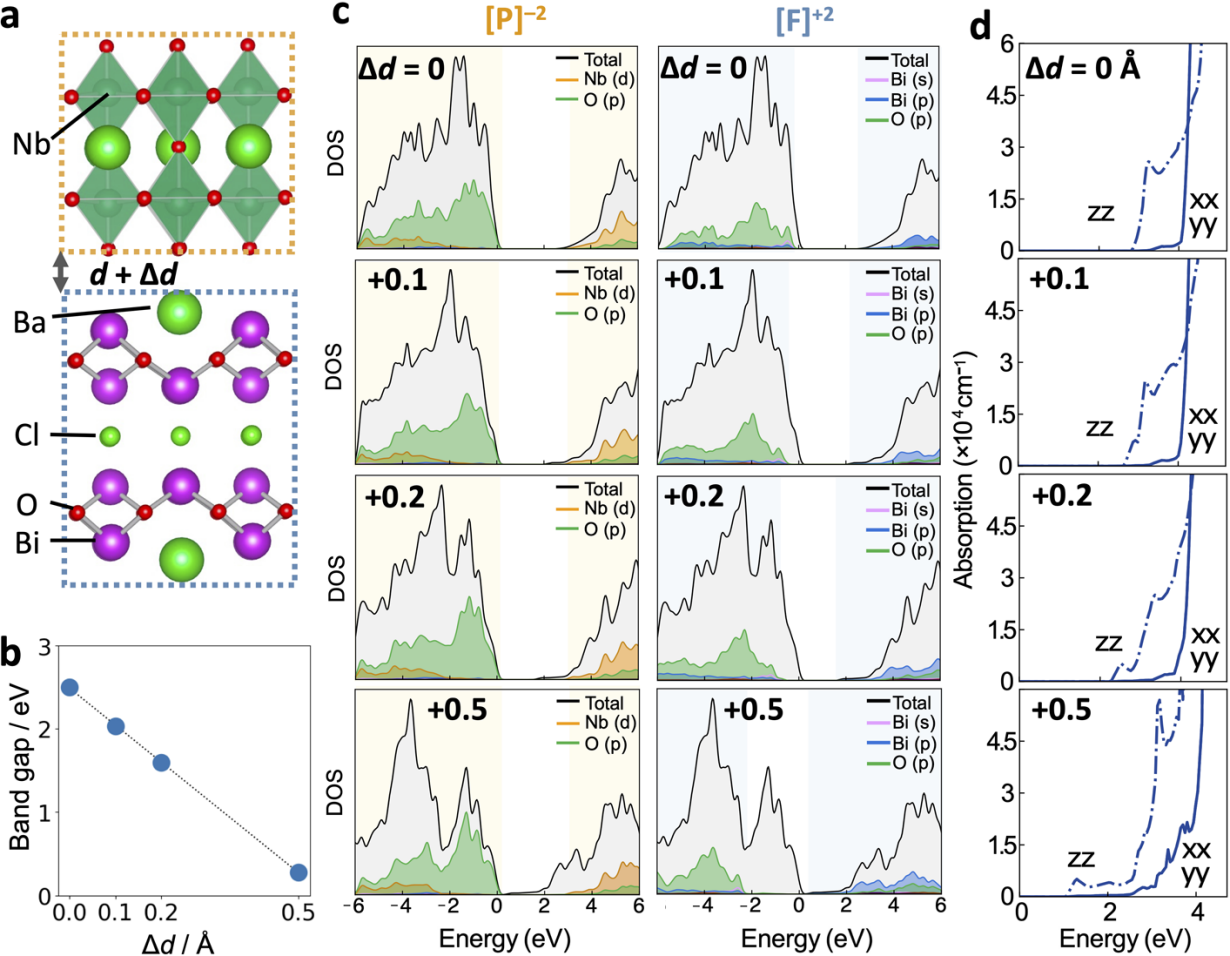
(a) Crystal structure of Ba_2_Bi_3_Nb_2_O_11_Cl highlighting the distance between charged perovskite [P]^+2^ and fluorite [F]^−2^ building blocks. (b) Calculated band gap change with interblock distance (Δd). (c) Electronic density of states (HSE06/DFT) of Ba_2_Bi_3_Nb_2_O_11_Cl with various interblock distances (Δ*d* = +0, +0.1, +0.2, +0.5 Å) with varying relative potential difference between the perovskite (orange) and fluorite blocks (blue). (d) Anisotropic optical absorption coefficients showing reduced transition energy along the *c* axis with an increased Δ*d* value.

Charged building blocks provide additional electrostatic interaction (Figure 1). To clarify this, we theoretically investigated the impact of interblock distance on the resulting electronic structure. Model structures were prepared by increasing the distance between [P]^−2^ and [F]^+2^ in Ba_2_Bi_3_Nb_2_O_11_Cl by Δ*d* (Å) (Figure 3a) from the ground state structure determined by DFT relaxation. The atomic positions were fixed within each block to exclude the relaxation effects. As shown in Figure 3b, as Δ*d* increases from 0 Å to +0.5 Å, the band gaps reduce from 2.45 to 0.27 eV, showing the opposite trend to the neutral vdW materials (Figure 1). This band gap reduction arises from the energetic approach of the O 2p upper valence band in the [P]^−2^ layer and the Bi 6p lower conduction band in the [F]^+2^ layer. The shift in the relative density of states between the [P]^−2^ and [F]^+2^ layers can be seen in Figure 3c and Figure S1. On the other hand, changes in the interblock distance do not have a significant influence on the intrablock interactions as the individual density of state components and band dispersion (effective masses) remain largely unchanged (Table S1). The band gap reduction is derived from a red shift in the z component of the optical absorption, although the intensity is relatively weak with α = 5 × 10^3^ cm^–1^ at 1.3 eV for Δ*d* = +0.5 Å (Figure 3d).

### Modular Electrostatic Model

The relative energy difference between the [F]^+2^ and the [P]^−2^ layers can be described by interblock electrostatics. While a conventional ion-based electrostatic model was tested, the site Madelung potentials alone could not describe the observed trends (Figure S2). Instead, we invoke a coarse-grained representation that regards each layer as charged–we confirmed that the interlayer charge redistribution is small for the perturbations we consider (Figure S3).

**Figure 4:**
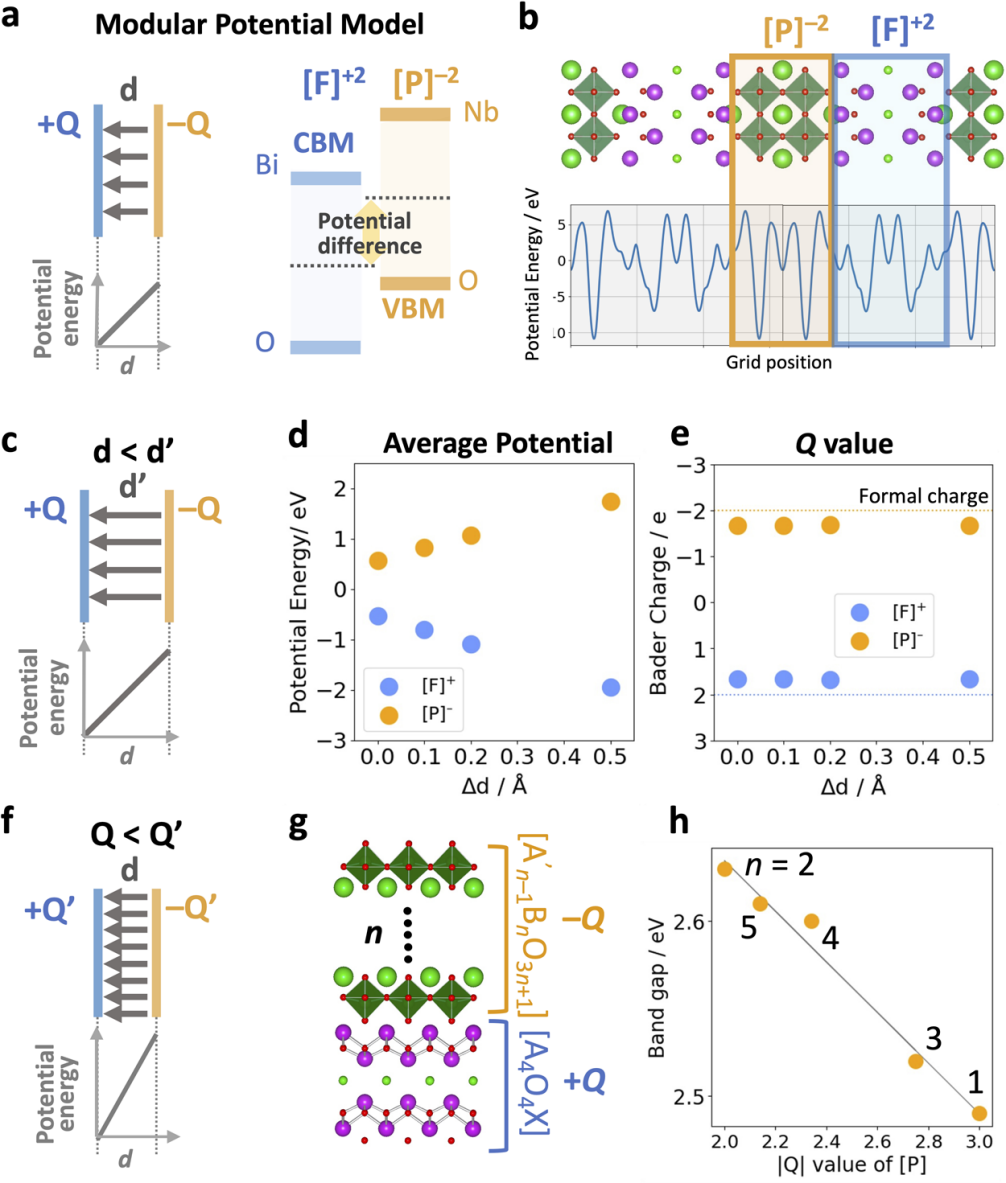
Modular electrostatic model to describe the electronic structure of Ba_2_Bi_3_Nb_2_O_11_Cl. (a) The charged fluorite [F]^+2^ and perovskite [P]^−2^ layers can be considered as a parallel plate capacitor with *Q* = 2. The [P]^−2^ at higher potential contributes to the upper valence band, while the [F]^+2^ at lower potential contributes to the lower conduction band. (b) 2D planar average potential in Ba_2_Bi_3_Nb_2_O_11_Cl from DFT calculations. (c–e) Effect of interlayer separation on the potential energy and charge. The larger the separation between blocks, the larger the potential energy difference. The value of *Q* is assessed by summing the Bader charge of each atom within the block. (f, h) Effect of increasing the layer charge. Experimental band gaps of Sillén–Aurivillius oxychlorides with different thickness (*n*) of the perovskite layer plotted versus the |*Q*| values of the perovskite layers in Bi_4_NbO_8_Cl, Ba_2_Bi_3_Nb_2_O_11_Cl, Bi_5_BaTi_3_O_14_Cl, Ba_2_Bi_5_Ti_4_O_17_Cl, and Ba_3_Bi_5_Ti_5_O_20_Cl.^42,37^

Adjacent layers with opposite charges produce a potential difference that can be described as a parallel plate capacitor with charge *Q* and layer separation *d* (Figure 4a):
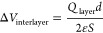
where ε is the effective dielectric permittivity and S is the surface area. The ability of the building blocks to screen the interlayer electric field is accounted for by ε. The factor of 2 in the denominator arises from the periodic boundary conditions (Figure S4). Here, the Δ*V*_interlayer_ represents a potential that stabilizes the +*Q* layer and destabilizes the −*Q* layer.

The potential difference between [F]^+2^ and [P]^−2^ directly explains the band separation in Ba_2_Bi_3_Nb_2_O_11_Cl shown in Figure 2c. It provides energy alignment of the [F]^+2^ and [P]^−2^ layers in Figure 4a, where the energetically higher [P]^−2^ contributes to the upper valence band, while the energetically lower [F]^+2^ contributes to the lower conduction band. As the interlayer separation is increased, the potential difference between [F]^+2^ and [P]^−2^ is enlarged (Figure 4c), which brings the CBM in [F]^+2^ and the VBM in [P]^−2^ closer and reduces the band gap, consistent with Figure 3.

To validate our model, we calculated the average potential of each layer block. The planar averaged electrostatic potentials from DFT were analyzed (Figure 4b). We further integrated them to calculate the average potential in each building block following the boundaries set by the orange and blue rectangles in Figure 4b. The average potential of the [P]^−2^ is higher than the [F]^+2^, and the difference grows with increasing interblock distance (Figure 4d), consistent with the modular potential model (Figure 4c). To assess the assumption of formal charges, we performed an analysis of the electron density from DFT by using the Bader formalism. This charge analysis confirmed that the increased distance negligibly affects the magnitude of *Q* (Figure 4e), owing to the weak through-space bonding (Figure S5). While the net charge is preserved, some charge redistribution does occur within each layer (Figure S6). The potential difference (Δ*V*) from the DFT results is 1.1 V, which is smaller than that estimated only from the coarse-grained model as the microscopic charge distribution and screening are neglected in the latter (Figure S4).

The modular electrostatic model suggests two controlling parameters: *d* and *Q*. In this family of compounds, the interblock distance can be modified by the alkaline earth cation size at the interface between [F]^+2^ and [P]^−2^ (i.e., Ba in the present case). The larger cation and thus a longer interblock distance increases the potential difference and reduces the band gap (Figure 4c). In fact, the experimental band gap of the Ba (1.61 Å) substitute (2.63 eV) is smaller than the Sr (1.44 Å) one (2.73 eV), though the A cation negligibly contributes to the frontier orbitals.^38^ Further band gap reduction can be achieved by combining larger alkaline cations Cs^+^ (1.88 Å) and La^3+^ (1.36 Å) as shown in Figure S7. Another parameter is the *Q* value; a larger *Q* results in a larger Δ*V* between the layers (Figure 4f). This effect on the band gap can be seen in the experimental values of several Sillén–Aurivillius oxychlorides with different thicknesses (*n*) of the perovskite layer (Figure 4g). These oxychlorides present different *Q* values depending on the constitutional element and thus *n*.^44,38^ As shown in Figure 4h, the larger |*Q*| value provides a smaller band gap owing to the increased potential difference.

The interblock electronic field even affects the oxygen potential within the perovskite block. The ordering of the oxygen site analyzed from the calculated electronic structure is O1 > O2 > O3 (Figure S8), where the farthest site from the interface with [F]^+2^ is the energetically highest. This oxygen order is the same as the upper valence band contribution (Figure 2b,c), which cannot be explained by the Madelung potential^47^ nor orbital interactions (Figures S2 and S5). Also in other Sillén–Aurivillius oxychlorides, the orbital contribution to the upper valence band gradually decreases from the center of the perovskite.^48^

### Extension to Other Layered Compounds

**Figure 5:**
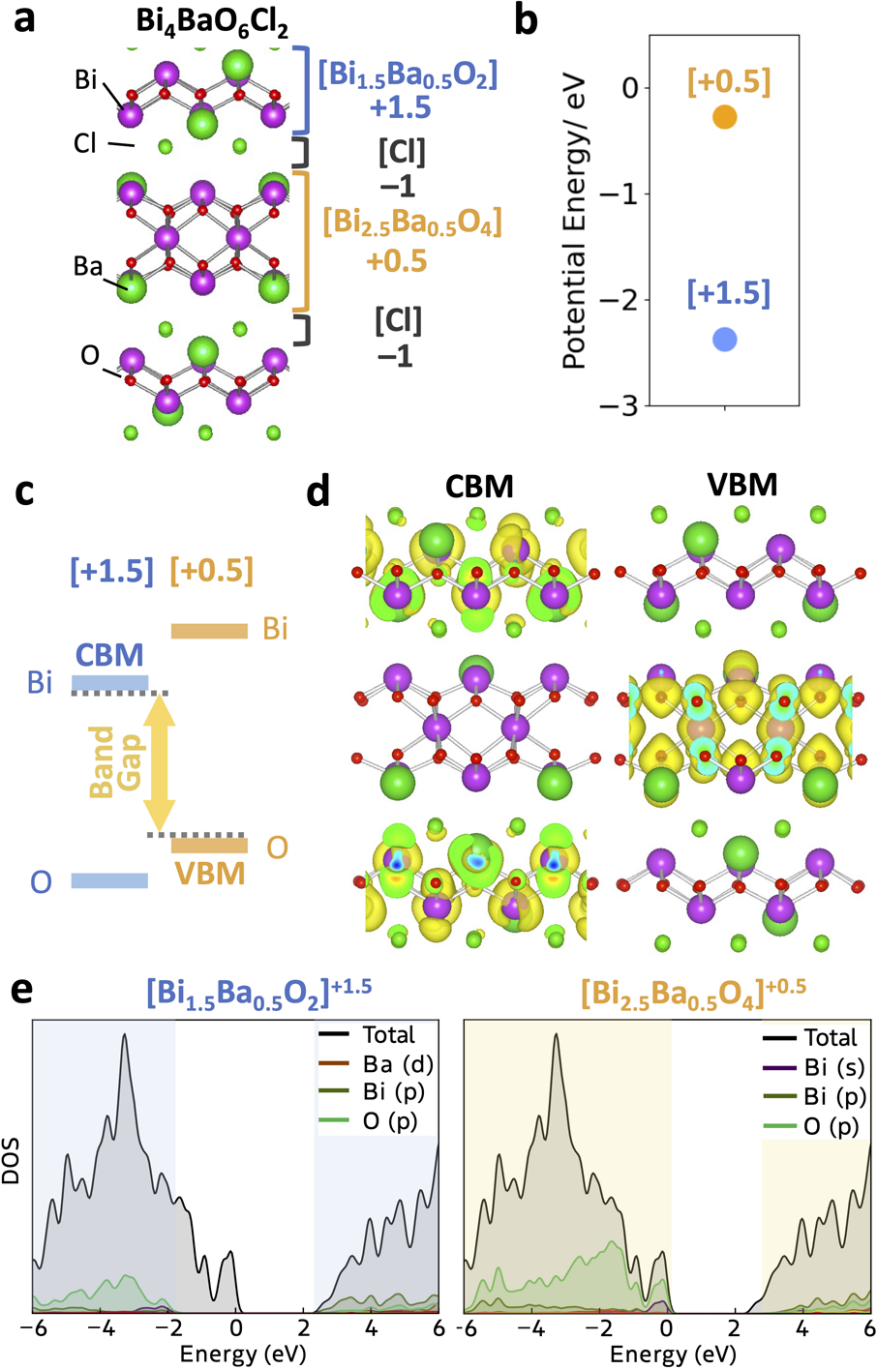
(a) Crystal structure of Sillén Bi_4_BaO_6_Cl_2_ as the stacking of [Bi_1.5_Ba_0.5_O_2_]^+1.5^, [Bi_2.5_Ba_0.5_O_4_]^+0.5^ layers with charge compensation Cl^–^ ions. (b) The average electrostatic energy of each block is estimated from the integrated potentials from density functional theory (HSE06). (c) Energetic band alignment of each block. (d) Electron density map of the conduction band maximum (CBM) and valence band minimum (VBM) at an isosurface value of 1.0 × 10^–3^ e/Å^3^. (e) Electron density of states (DOS), with a CBM on the more positive block and a VBM on the less positive block.

Beyond the Sillén–Aurivillius series, this model describes the electronic structures of other charged layered materials. The first example is a Sillén-type Bi_4_BaO_6_Cl_2_ photocatalyst.^49^ The double fluorite [Bi_1.5_Ba_0.5_O_2_]^+1.5^ and triple fluorite [Bi_2.5_Ba_0.5_O_4_]^+0.5^ layers are charge compensated by negatively charged [Cl]^−^ ions (Figure 5a). The distinct positive values (i.e., + 1.5 and +0.5) impact the electrostatic potential of these layers, where the less positive [Bi_2.5_Ba_0.5_O_4_]^+0.5^ layer is less stabilized and higher in potential than [Bi_1.5_Ba_0.5_O_2_]^+1.5^ as shown in Figure 5b. This potential difference dictates the electronic alignment of these layers (Figure 5c), leading to the spatial separation of the upper valence band on [Bi_2.5_Ba_0.5_O_4_]^+0.5^ and the lower conduction band on [Bi_1.5_Ba_0.5_O_2_]^+1.5^ (Figure 5d). Additional effects are seen in the calculated DOS (Figure 5e) where the density components from each layer in the valence and conduction bands are aligned as in Figure 5c. Even when the two layers are composed of the same elements, the same sign of charge, and a similar structural character, the electrostatic potential differences still play a critical role.

The second example is a Ruddlesden–Popper oxide LaSrAlO_4_. Balachandran and Rondinelli theoretically demonstrated the large band gap reduction of LaSrAlO_4_ by changing the cation order.^50,51^ LaSrAlO_4_ is composed of stacked single-perovskite layers, where the B site is Al and the A site is La or Sr. As in the polar discontinuity, as mentioned above, LaSrAlO_4_ can be decomposed into three planes: [LaO]^+^, [SrO]^0^, and [AlO_2_]^−^. Three model structures were considered, [LaO|AlO_2_|SrO][LaO|AlO_2_|SrO] (no. 1), [LaO|AlO_2_|SrO][SrO|AlO_2_|LaO] (no. 2), and [LaO|AlO_2_|LaO][SrO|AlO_2_|SrO] (no. 3), where the last structure showed the significantly reduced band gap. Importantly, a significant band gap reduction was observed even without the ionic relaxation.

**Figure 6:**
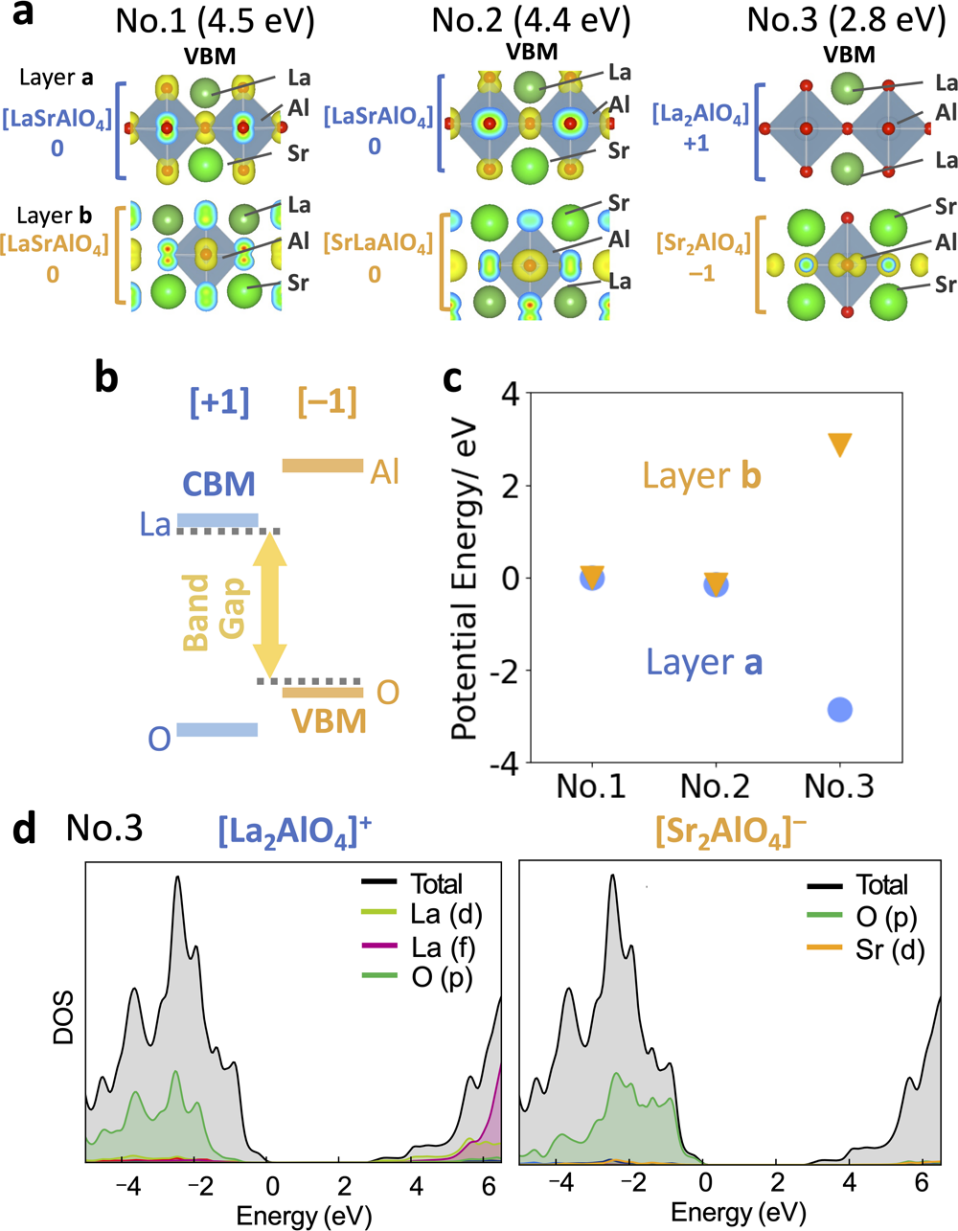
(a) Three model crystal structures of Ruddlesden–Popper LaSrAlO_4_ with different A site cation (La and Sr) ordering. No. 1, no. 2, and no. 3 correspond to [LaSrAlO_4_]^0^ + [LaSrAlO_4_]^0^, [LaSrAlO_4_]^0^ + [SrLaAlO_4_]^0^, and [La_2_AlO_4_]^+^ + [Sr_2_AlO_4_]^−^, respectively. An electron density map of the valence band maximum (isosurface values of 5 × 10^–3^, 5 × 10^–3^, and 5 × 10^–4^ e/Å^3^, respectively.) is projected on each structure (HSE06/DFT). (b) Energetic band alignment of each block. (c) Average potential energy of each block, which is estimated by summing the planar potential within the block. (d) Electron density of states of each charged building block for no. 3.

From a modular perspective, these structures can be described as [LaSrAlO_4_]^0^ + [LaSrAlO_4_]^0^, [LaSrAlO_4_]^0^ + [SrLaAlO_4_]^0^, and [La_2_AlO_4_]^+^ + [Sr_2_AlO_4_]^–^, by considering the net composition within each block (Figure 6a). Only the no. 3 model structure is composed of the charged layers, which generates the internal electric fields where the negative [Sr_2_AlO_4_]^−^ layer is higher in energy than the positive [La_2_AlO_4_]^+^ based on our modular electrostatic model (Figure 6b). The potential between these layers in the no. 3 structure is calculated to be different, while the potential difference between the neutral-charged blocks in no. 1 and no. 2 is negligible owing to the absence of the interblock electric field (Figure 6c). Due to this effect, the upper valence band of no. 3 is composed of O 2p in [Sr_2_AlO_4_]^−^ consistent with Figure 6b, while the wave functions in no. 1 and no. 2 are delocalized across the two layers (Figure 6a). Our model well describes the narrower band gap of no. 3 and its characteristic electronic structure with the spatially separated upper valence band (Figure 6d). It is noteworthy that, given that the energy difference between these structures is less than 50 meV/atom (Figure S9), the cation ordering can be a plausible strategy for band gap tuning.^52^

Besides the layered materials investigated here, there are many families of layered materials that can be regarded as stacked charged modules. Some of these are summarized in Table 1. For example, the water-splitting photocatalyst Y_2_Ti_2_S_2_O_5_ can be depicted as stacked [Y_2_S_2_]^2+^ and [Ti_2_O_5_]^2–^. The relatively stable S 3p and the destabilized O 2p, which are the keys to its overall water-splitting ability,^53^ can be described by our modular potential model. Here, S 3p in the positive [Y_2_S_2_]^2+^ is stabilized while O 2p in the negative [Ti_2_O_5_]^2–^ is destabilized (Figure S10). Ba_3_*M*O_5_Cu_2_*Ch*_2_ (M = Sc, In and *Ch* = S, Se) has also an upper valence band from the negative [Cu_2_*Ch*_2_]^−2^ module and a lower conduction band from the positive [Ba_3_*M*_2_O_5_]^+2^ module, as expected from the present model.^54^ The BiS_2_-based superconductors also can be regarded as the staked layer module.^55^ We note that the present model assumes the electronic distribution contained within each block and may not be appropriate for cases in which electrons are localized on a specific element. Although NaCoO_2_ can be regarded as [Na]^+^ and [CoO_2_]^−^ layer, the valence electrons are localized on the 3 d orbitals in Co^3+^, and thus the modular model failed for this case (Figure S11). Despite such exceptions, the modular perspective and the resultant electrostatic differences well describe the electronic structure trends of complex layered materials.

**Table 1:** Examples of Layered Materials that Can Be Regarded as Stacked Charged Modules

compound	positive block	negative block	application
Y_2_Ti_2_S_2_O_5_	[Y_2_S_2_]^+2^	[Ti_2_O_5_]^−2^	photocatalyst^53^
Ba_3_Sc_2_O_5_Cu_2_S_2_	[Ba_3_Sc_2_O_5_]^+2^	[Cu_2_S_2_]^−2^	photocatalyst,^54^ transparent p-type conductor^56^
Sr_2_Cu_2_ZnO_2_S_2_	[Sr_2_ZnO_2_]^+2^	[Cu_2_S_2_]^−2^	transparent p-type conductor^57^
BiOBiS_2_	[Bi_2_O_2_]^+2^	[Bi_2_S_4_]^−2^	spin electronics^58^
Bi_2_WO_6_	[Bi_2_O_2_]^+2^	[WO_6_]^−2^	photocatalyst,^59^ spin electronics^60^
BaBi_2_Nb_2_O_9_	[Bi_2_O_2_]^+2^	[BaNb_2_O_7_]^−2^	ferroelectrics^61^
LaOFeAs	[La_2_O_2_]^+2^	[Fe_2_As_2_]^−2^	superconductor^62^
BaFe_2_As_2_	[Ba]^+2^	[Fe_2_As_2_]^−2^	superconductor^63^
Y_2_O_2_Bi	[Y_2_O_2_]^+2^	Bi^2–^	superconductor^64^
NaCoO_2_	[Na]^+^	[CoO_2_]^−^	ion conductor,^65^ superconductor^66^

## Conclusions

The extension of established chemical principles to modular crystals with many degrees of freedom can be challenging. In this study, we introduced a coarse-grained electrostatic model to describe the electronic trends in modular materials based on charged building blocks. The negatively and positively charged layer units produce internal electric fields with an electrostatic potential difference between them that can shift the resulting electronic band structures.

The magnitude of the electronic band edge shifts can be tuned by the interblock distance and the charge values, providing a crystal chemical lever to control electronic properties. Substitution of atoms near the interblock boundary or module itself can be a practical approach as demonstrated in layered oxyhalides. Given that the present modular perspective is general and based on electronic delocalization, the hybrid organic–inorganic layered halide perovskites composed of a negatively charged perovskite layer and interlayer cationic molecules, e.g., Dion–Jacobson type (4AMPY)(MA)Pb_2_I_7_^67^ can be promising extensions. A modular perspective may help us to understand and design such complex materials with tailored electronic properties.

## Computational Methods

The initial crystal structure for the Sillén–Aurivillius compound Ba_2_Bi_3_Nb_2_O_11_Cl was taken from a previous X-ray diffraction refinement.^40^ All electronic structure calculations were performed using density functional theory (DFT) within periodic boundary conditions through the Vienna ab initio simulation package (VASP).^43^ The projector-augmented-wave (PAW) method was employed. Calculations for Sillén–Aurivillius oxyhalides, Sillén Bi_4_BaO_6_Cl_2_, Ruddlesden–Popper LaSrAlO_4_ were carried out using the Heyd–Scuseria–Ernzerhof hybrid functional (HSE06).^42^ For other materials, the Perdew–Burke–Ernzerhof revised for solid (PBEsol)^68^ formulation of the generalized gradient approximation (GGA) was employed as the exchange-correlation functional. Note that we confirmed that the characteristics in the electronic structure of Ba_2_Bi_3_Nb_2_O_11_Cl remain even with the PBEsol functional (Figure S12). The plane-wave energy cutoff energy and Γ-centered *k*-point mesh were sequentially increased using the vaspup2.0 package^69^ until the total energies from static calculations were converged to within 1 meV/atom. The given values were 600 eV and 4 × 4 × 1 for Ba_2_Bi_3_Nb_2_O_11_Cl, 600 eV and 2 × 2 × 1 for Bi_4_BaO_6_Cl_2_, 600 eV and 8 × 8 × 2 for LaSrAlO_4_. The atomic positions were optimized until the Hellman–Feynman forces on each atom were below 0.01 eVÅ^–1^. The energy convergence criterion was set to 10^–6^ eV. A

Electronic band structure diagrams were generated and analyzed using the sumo package.^70^ Crystal orbital Hamilton population (COHP) was calculated using the LOBSTER package.^71^ The planar average potential was analyzed by the MacroDensity package.^72^ The average potential within a layer was estimated by summing the planar potential within the layer unit, where the middle point between the two layers was set as the starting and ending points of the summation. For the case of the model structure with increased interlayer distance in Ba_2_Bi_3_Nb_2_O_11_Cl, the summation range was fixed as in the original structure.

## Supporting Information Available

Additional theoretical calculation results (PDF)
