# Supplementary material for: Electrostatic Control of Electronic Structure in Modular Inorganic Crystals
Source: J Am Chem Soc. 2024 Dec 19;147(1):821–9. doi: 10.1021/jacs.4c13637 (PMC11726560; doi:10.1021/jacs.4c13637)
Supplement: Supplementary file 1 — ja4c13637_si_001.pdf [file ja4c13637_si_001.pdf]

# Supporting Information

# Electrostatic Control of Electronic Structure in Modular Inorganic Crystals

Kanta Ogawa[1,†] and Aron Walsh[1]

[1]Department of Materials, Imperial College London, London SW7 2AZ, UK
[†]Present address: Materials and Structures Laboratory, Institute of Integrated Research, Institute of Science Tokyo, R3-7, 4259 Nagatsuta, Midori-ku, Yokohama 226-8501, Japan

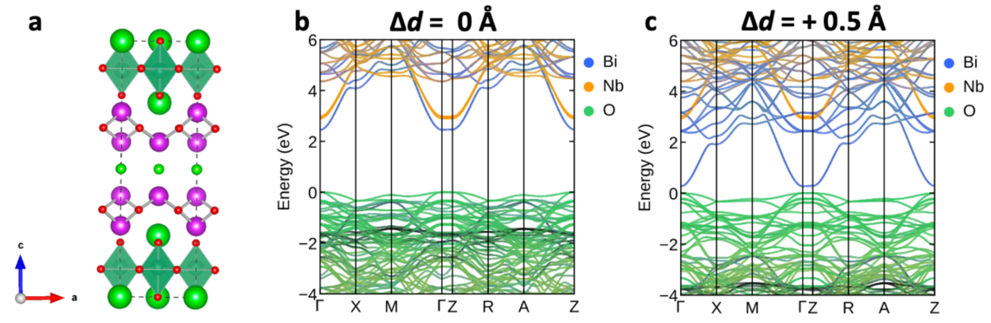


Figure S1. (a) The crystallographic unit cell (*P*4/*mmm*) and (b,c) electronic band structures of $Ba_2Bi_3Nb_2O_{11}Cl$ with Δ*d* = (b) 0 and (c) 0.5 Å.

Table S1. The effective masses calculated by parabolic fitting of the band dispersion.

| | | in-plane | out-of-plane |
|---|---|---|---|
| $\Delta d = 0$ Å | $m_e^*/m_0$ | 0.26 | 2.79 |
| | $m_h^*/m_0$ | 1.84 | – |
| $\Delta d = 0.5$ Å | $m_e^*/m_0$ | 0.25 | 2.69 |
| | $m_h^*/m_0$ | 1.89 | – |

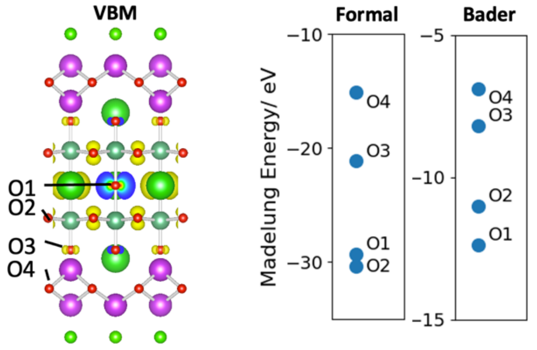


Figure S2. Madelung potential of each oxygen site. O1, O2, O3 are included in the perovskite block, while O4 is in the fluorite block. The formal charge (i.e., −2) or the Bader charge calculated from DFT results was employed.

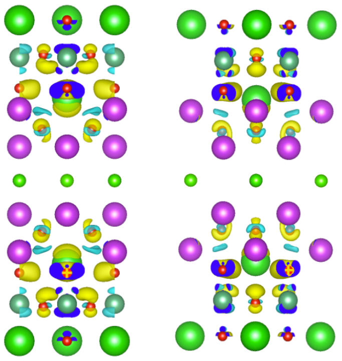

Figure S3. Charge density difference between the bulk and each charged block. Charge redistribution accompanied by the stacking is within each block.

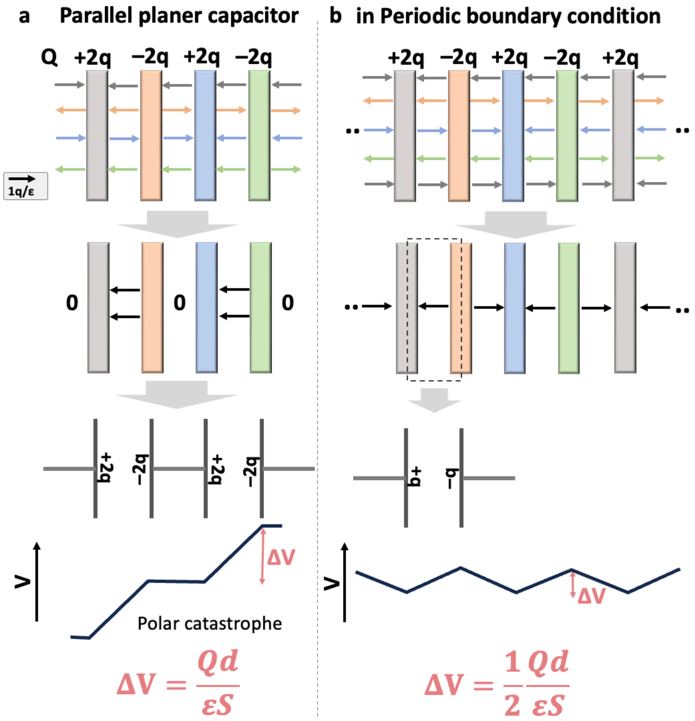


Figure S4. Parallel planar model with the periodic boundary condition. For parallel plate capacitors without the periodic boundary condition, the potential in the system can be described as in (a). Here, each of the four planes with $Q$ value ($2q$) facing each other produces two arrows (each arrow represents 1q/ε electric fields) based on Gauss's row. The four planes are described by two capacitors in series, where each capacitor has $\Delta V = Qd/\varepsilon S$, with $Q = 2q$, interplate distance $d$, permittivity ε, and surface area of the plane S. The total $\Delta V$ is provided by the sum of the $\Delta V$ of each capacitor, and increasing the number of capacitors results in an increased $\Delta V$ across the whole system. However, this model does not include the periodic boundary condition. Therefore, the infinite number of planes results in the divergence of $\Delta V$ (as in the polar catastrophe), being not an adequate model for the periodic crystals. With the periodic boundary condition, i.e., $\phi(0)=\phi(\infty)$, the first and last planes should be the same.

In other words, the green plane in (a) should interact with the first grey plane as in (b). In this case, $\Delta V$ is provided by $Qd/2\varepsilon S$ (one can conclude this just by counting the number of the arrow) with alternative increase and decrease of $\Delta V$ without divergence. By regarding $Q$ is the charge of a layer in a unit cell, S is the area of the a-b plane, $\varepsilon = \varepsilon_0$ assuming no medium between the layers, $\Delta V$ is calculated to be 14 V only from the structural parameter. The larger value than the DFT values (1.1 V) may be derived from the non-consideration of the charge distribution along the units. The charge distribution can provide a smaller $Q$ value and the dielectric constant of the material should be considered, which is relatively large for this material.

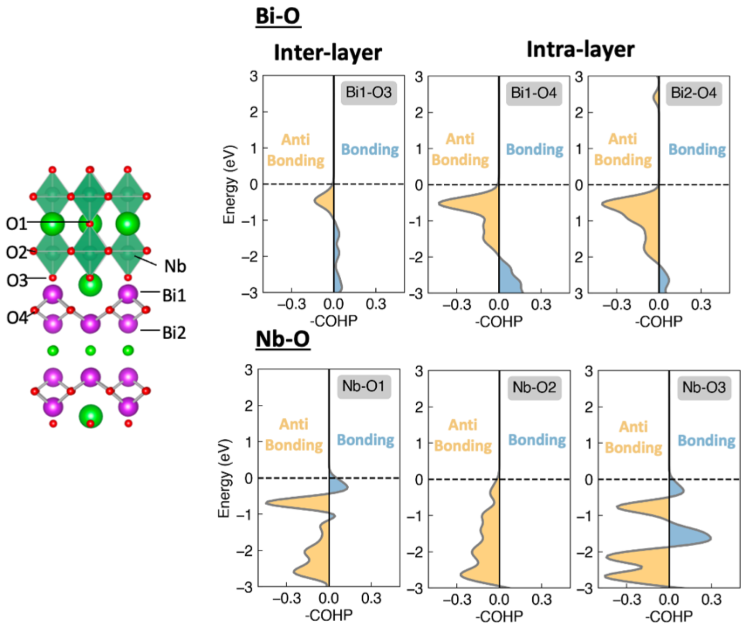


Figure S5. Crystal orbital Hamilton populations (COHP) for Bi1 and O3, O4 and Nb and O1, O2, O3 in the perovskite layer of $Ba_2Bi_3Nb_2O_{11}Cl$. The orbital interaction may not be the origin of the oxygen contribution order to the VBM because of the similar interaction manner of O1 and O3 with Nb around the VBM.

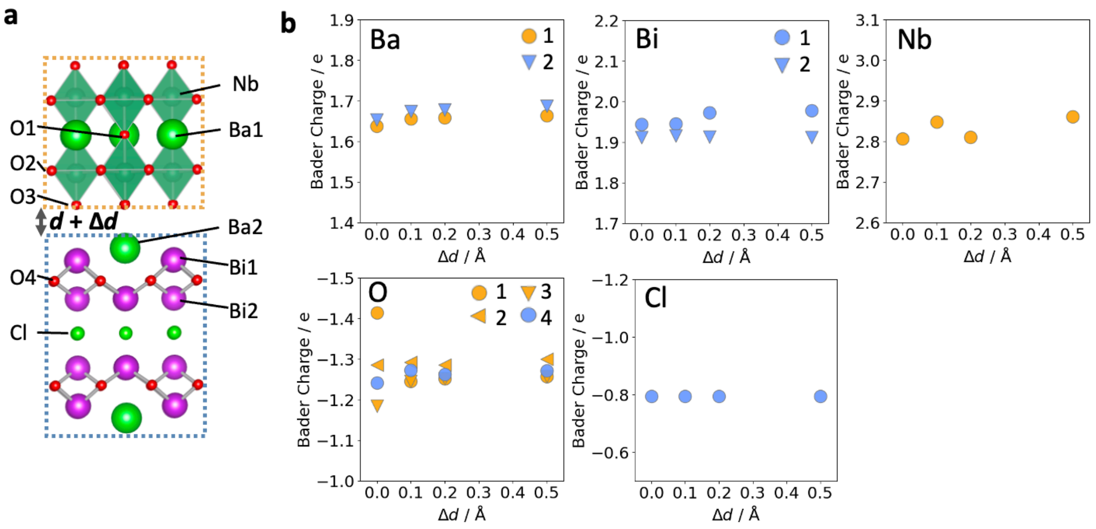


Figure S6. (a) Crystal structure of $Ba_2Bi_3Nb_2O_{11}Cl$ and (b) the effect of Bader charge of each element by the inter-layer distance. The changes of the Bader are relatively small (a few percent) except for oxygen. The Bader charge of O1 at the center of the perovskite layer is increased by increased Δ*d* because the O1 contributing to the VBM is susceptible to the increased Δ*V*. Judging from the reduced charge of O3 (and the no-change in the total charge of each layer), these results suggest that the charge redistribution by the change of the inter layer distance occurs within each layer.

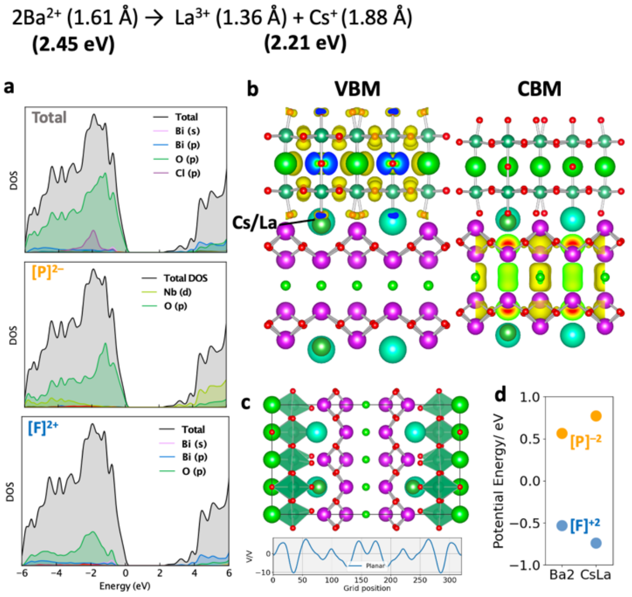


Figure S7. (a) Electron density of states (HSE06) of $LaCsBi_3Nb_2O_{11}Cl$. (b) Electron density map of the valence band maximum and conduction band minimum at isosurface values of $1 \times 10^{-4}$ and $5 \times 10^{-5}$ e/$Å^3$, respectively. (c) 2D planar average potential. (d) The average potential energy of $[F]^{+2}$ and $[P]^{-2}$ blocks estimated by summing the planar potential within the block.

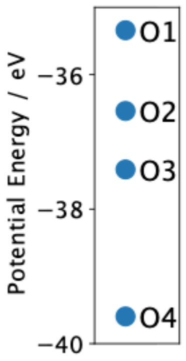


Figure S8. The potential energy of each oxygen site analyzed from the calculated electronic structure of $Ba_2Bi_3Nb_2O_{11}Cl$.

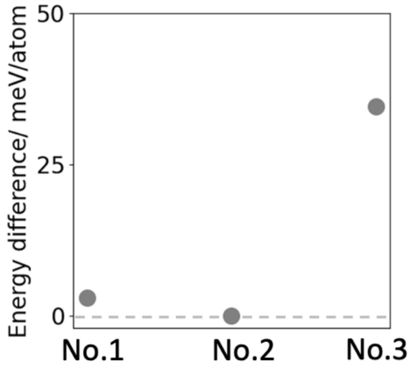


Figure S9. Total energy difference between the three model crystal structures of Ruddlesden-Popper $LaSrAlO_4$ in Figure 6.

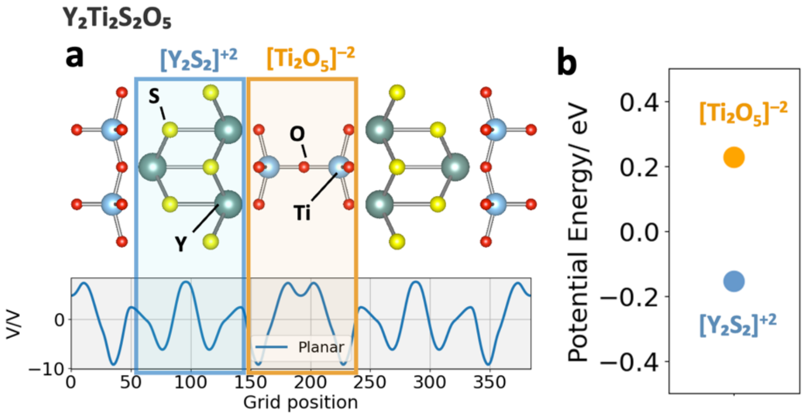


Figure S10. (a) Modular view of $Y_2Ti_2S_2O_5$ and 2D planar average potential. (b) The average potential energy of $[Y_2S_2]^{+2}$ and $[Ti_2O_5]^{-2}$ blocks, which is estimated by summing the planar potential within the block.

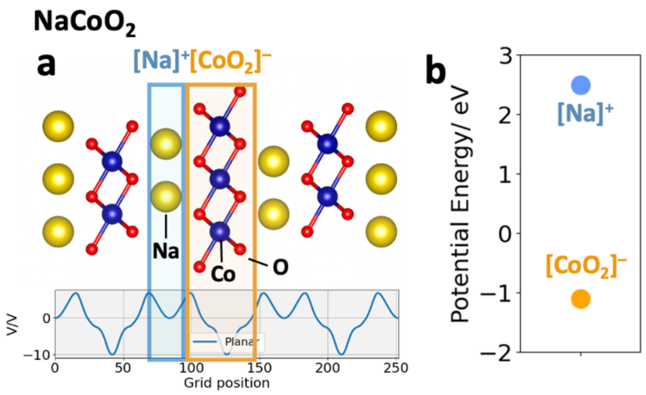


Figure S11. (a) Modular view of $NaCoO_2$ and 2D planar average potential. (b) The average potential energy of $[Na]^+$ and $[CoO_2]^-$ blocks, estimated by summing the planar potential within the block. The modular model is not appropriate in this case because of the strong ionic character with localized valence electrons on 3d orbitals of $Co^{3+}$.

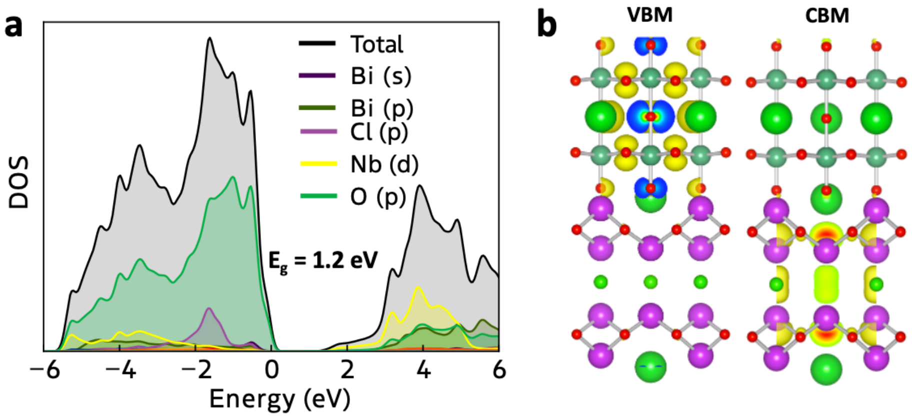


Figure S12. (a) Electronic density of states (PBEsol/DFT) and (b) electron density map of the valence band maximum and conduction band minimum of $Ba_2Bi_3Nb_2O_{11}Cl$.
